# COVID-19 and Ventilator-Associated Event Discordance

**DOI:** 10.1017/ash.2021.82

**Published:** 2021-07-29

**Authors:** Kelly Cawcutt, Mark Rupp, Lauren Musil

## Abstract

**Background:** The COVID-19 pandemic has challenged healthcare facilities since its discovery in late 2019. Notably, the subsequent COVID-19 pandemic has led to an increase in healthcare-acquired infections such as ventilator associated events (VAEs). Many hospitals in the United States perform surveillance for the NHSN for VAEs by monitoring mechanically ventilated patients for metrics that are generally considered to be objective and preventable and that lead to poor patient outcomes. The VAE definition is met in a stepwise manner. Initially, a ventilator-associated condition (VAC) is met when there an increase in ventilator requirements after a period of stability or improvement. An IVAC is then met when there is evidence of an infectious process such as leukocytosis or fever and a new antimicrobial agent is started. Finally, possible ventilator-associated pneumonia (PVAP) is met when there is evidence of microbial growth or viral detection. Since the beginning of the COVID-19 pandemic, our hospital has seen an increase in VAEs, which is, perhaps, not unexpected during a respiratory illness pandemic. However, the NSHN definitions of VAE, and PVAP in particular, do not account for the novelty and nuances of COVID-19. **Methods:** We performed a chart review of 144 patients who had a VAE reported to the NHSN between March 1 and December 31, 2020. **Results:** Of the 144 patients with a VAE reported to NHSN, 39 were SARS-CoV-2 positive. Of the 39 patients, 4 patients (10.25%) met the NHSN PVAP definition due to a positive SARS-CoV-2 PCR that was collected in the prolonged viral shedding period of their illness (< 90 days). One of the four patients also had a bacterial infection in addition to their subsequent positive COVID-19 result. All these patients were admitted to the hospital with a COVID-19 diagnosis and their initial PCR swab was performed upon admission. **Conclusions:** We believe that the PVAP definition was inappropriately triggered by patients who were decompensating on the ventilator due to a novel respiratory virus that was present on admission. Early in the pandemic, frequent swabbing of these patients was performed to try and understand the duration of viral shedding and to determine when it would be safe to transfer patients from isolation after prolonged hospitalization. The NSHN definition should take into consideration the prolonged viral shedding period of COVID-19 and natural history of the illness, and subsequent COVID-19 testing within 90 days of an initial positive should not require classification as a hospital-acquired PVAP.

**Funding:** No

**Disclosures:** None

